# Supplementary material for: Predicting visual function by interpreting a neuronal wiring diagram
Source: Nature. 2024 Oct 2;634(8032):113–23. doi: 10.1038/s41586-024-07953-5 (PMC11446822; doi:10.1038/s41586-024-07953-5)
Supplement: Supplementary file 6 — CRF and ERF predictions for individual TmY4 and TmY9 cells. Analogous to Supplementary Data 3, but for TmY target types. Shown are the top four monosynaptic pathways, the strongest pathway passing through each of the top ten intermediary types (ranking from Extended Data Fig. 7), and the trisynaptic pathway Tm1–TmY–Dm3–TmY (see the section entitled Prediction of spatial normalization). [file 41586_2024_7953_MOESM6_ESM.zip › DataS4/TmY9qΓèÑ/720575940611741282.pdf]

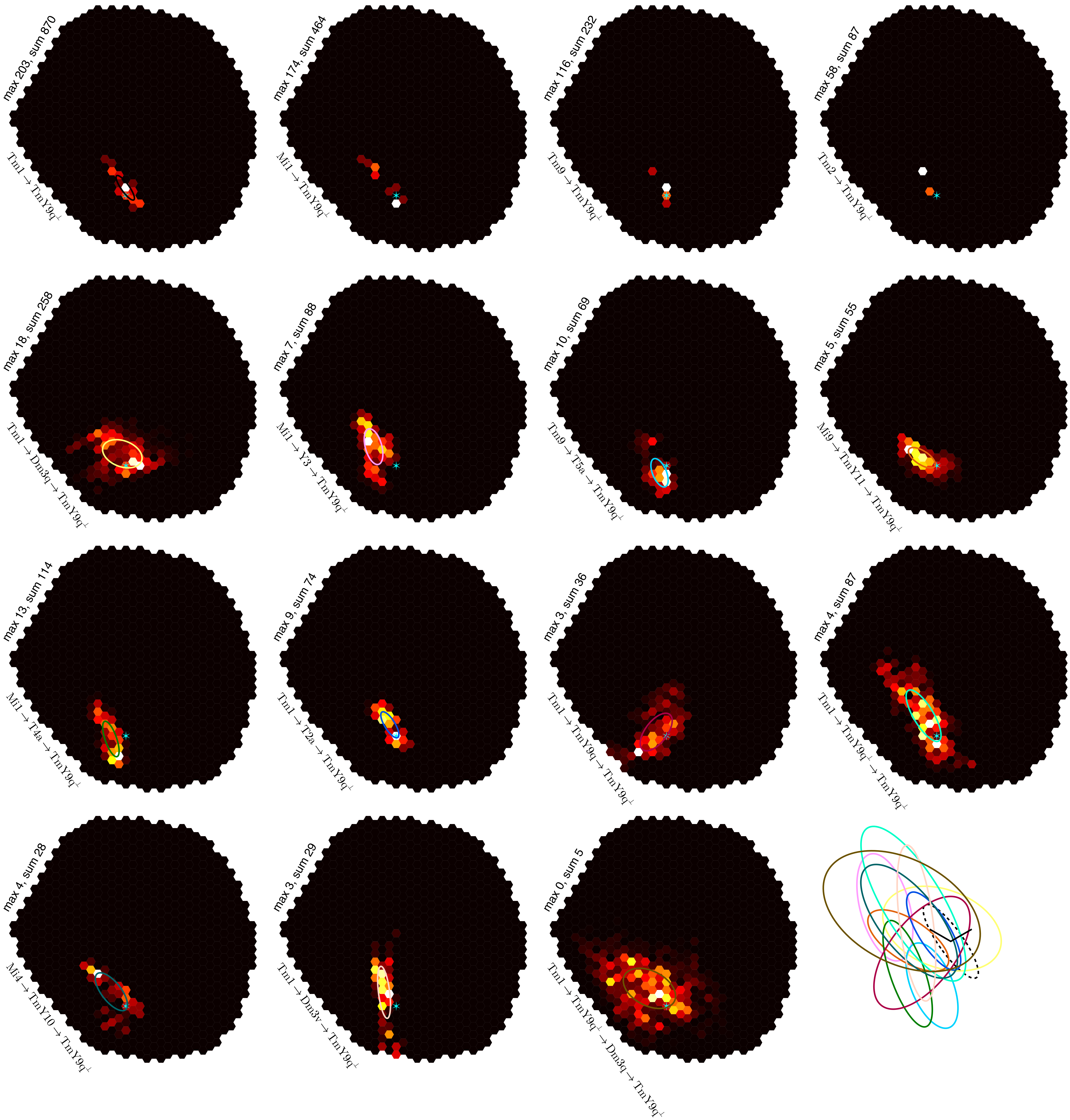
max 203, sum 870
Tm1 → TmY9q⊥
max 174, sum 464
Mi1 → TmY9q⊥
max 116, sum 232
Tm9 → TmY9q⊥
max 58, sum 87
Tm2 → TmY9q⊥
max 18, sum 258
Tm1 → Dm3q → TmY9q⊥
max 7, sum 88
Mi1 → Y3 → TmY9q⊥
max 10, sum 69
Tm9 → T5a → TmY9q⊥
max 5, sum 55
Mi9 → TmY11 → TmY9q⊥
max 13, sum 114
Mi1 → T4a → TmY9q⊥
max 9, sum 74
Tm1 → T2a → TmY9q⊥
max 3, sum 36
Tm1 → TmY9q → TmY9q⊥
max 4, sum 87
Tm1 → TmY9q⊥ → TmY9q⊥
max 4, sum 28
Mi4 → TmY10 → TmY9q⊥
max 3, sum 29
Tm1 → Dm3v → TmY9q⊥
max 0, sum 5
Tm1 → TmY9q⊥ → Dm3q → TmY9q⊥
